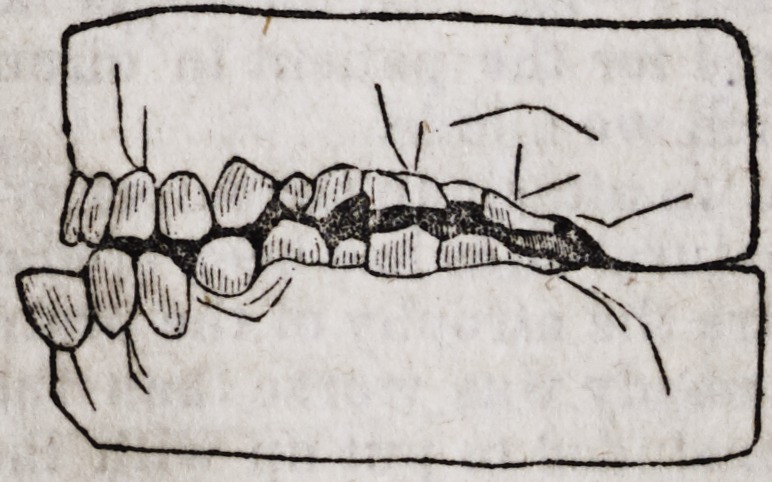# From La Lancette Francaise, Paris, Saturday, 14th November, 1840

**Published:** 1841

**Authors:** J. J. Greenwood

**Affiliations:** 71 Warren-st., New-York.


*From La Lancette Francaise, Paris, Saturday, 14th November,* 1840.

TRANSLATED BY J. J. GREENWOOD, 71 WARREN-ST., NEW-YORK.

1st. The anomalous developments of the anterior portion of the superior maxillary bone,—the great obliquity and prominence of the incisive teeth of that bone, and the remedy after two months' treatment. By Mr. Brewster, Surgeon Dentist.

2d. The retraction, on the same subject, of the upper lip: the section of the myrtiform muscle, and the cure.

Miss S., a young English lady of about sixteen years of age, of a strong constitution, and enjoying excellent health, was presented to Mr. Brewster by her family under the following circumstances: The anterior teeth of the superior dental arch presented a considerable obliqueness in front. This obliquity was so positive, that the teeth offered to view almost a horizontal position. The alveolar process in all its incisive portion, so projected itself in front that the cutting edges of the inferior incisors represented upon the accompanying figure by the punctuated lines *a a*, was removed almost two centimetres (about 5-8 of an inch) from the cutting edges of the corresponding superior incisors of the upper jaw.

This so rare a deviation of the alveolar circle (for it is almost never observed even in any case of the projections of the teeth, where for the most part the crowns are carried forward, and the roots themselves preserving three nominal positions) gave the jaw so unnatural an appearance that the visage had the most repulsive aspect, as can easily be deduced from the figures, which are the exact transcripts of the models taken from the patient.

This deformity presented other inconveniences, viz: the teeth could not come in contact, and the result was an enormous opening, which was an obstacle to masticating alimentary substances, and to the proper pronunciation of a great number of words.

This projection of the maxillary bone and the teeth, had produced by its long standing, a lifting up of the upper lip, amounting almost to an overlapment, (or folding upon itself) strongly sustained and bound under the fibrous cartilage of the nose.

The myrtiform muscle had entirely lost its mobility, so much so that the lip, although perfectly developed otherwise, could in no wise fulfil the functions to which it is destined during mastication, and while pronouncing a great many of the consonants. In a word this young lady could but poorly perform the operation of mastication, and was much more deficient in that of pronunciation. The palatine vault somewhat straightened and laterally devious, added still to these difficulties already too obvious. Under all other points of view, Miss S. was of an agreeable form; her features regular; her eyes beautiful and expressive; and notwithstanding these combined advantages—her education, her agreeable disposition, and the rich reversion which awaited her as the sole heiress of an immense fortune, she was thus destined to bear up against the perpetual mortification and endurement of this hideous infirmity.

Under these afflicting circumstances, her parents who were continually travelling for many years in the different countries of Europe, did not fail to apply to the most distinguished dentists of those places they were visiting; and those especially of the great cities of England, of France and of Italy have no doubt a recollection of having seen Miss S. All those practitioners were of the common opinion, and fully agreed in the impossibility of giving any assistance except by the operation which consisted in nothing less than the extraction of all the incisor and canine teeth: that is to say, removing six sound and healthy organs, and which being placed in order, and occupying their *proper situations* would of themselves become beautiful ornaments.

Far be it from us to reproach in the least those who have given such advice, unfortunate as it must have been to the parents of this young lady. On the contrary, we would with pleasure rather render that homage due to their talents, and especially to the many of their individual and beautiful operations; but it is not less our duty to point out a fact which has already been reported by other practitioners—for this improper advice is often the sole resort which may be prepared for the patient in circumstances less grave and serious than those to which we allude.

It was likewise advised to attach to the superior dental arch a plate with artificial teeth, after the natural ones had been eradicated; and it was expected by this means to procure the atrophy of the protrusion of the edges of the alveolar process. The remedy was worse than the evil complained of, and the parents of the lady concluded to put up with the lot which had been assigned to them.

In passing through Paris, some months since, on their route to Naples, they consulted Mr. Brewster, who concluded that it would be most proper and necessary to preserve the teeth in remedying the deformity; and he undertook the task notwithstanding the difficulties which presented themselves, although the smallness of the teeth, and the little hold which offered for the necessary operations and affixtures rendered the chances of success still less. Without entering into technical details, altogether useless here, we will but mention the means employed by Mr. Brewster, which were of the following character.

A gold plate was fitted to the inner circle of the alveolar process upon the palatine vault: this plate supported a sett of spiral springs, which were attached to the disarranged organs, and were intended to exercise upon them by their contraction a gradual drawing of the teeth inwards. These means succeeded beyond his most sanguine hopes; for in a very short time (less than three months) the teeth altered their position, and the edges of the alveolar process retook their proper form and natural direction so much so that the mouth of Miss S. has, we can now say, become regulated, and much more handsome than most young ladies of her age.

By the following figures, you will be better enabled to judge than words can express to you, the great benefits derived from this method of treatment. The alveolar arch, presents a proper curve, and the teeth of both jaws meet at the ordinary point of junction.

The patient's projection in front has disappeared.

The cavity formed by the elongated separation of the two arches has become filled up.

And in a few days Miss S. will be enabled to eat with facility, and to make herself much more distinctly understood.

Although this wonderful change had taken place in Miss S. as respected the teeth, still the retraction of the lips remained, notwithstanding the recent advantages obtained. M. Blandin, who was consulted by Mr. Brewster, was of the opinion that by an incision made into the myrtiform muscle, and a proper compression adapted, the second infirmity might be remedied. This small but important operation was performed by the Surgeon we have named above, assisted by Dr. Olliffe.

The muscle of the lip was seized by pincers, and the section made by curved scissors. Two eliptical incisions were performed on the mucous membrane of each side of the corner of the lip—this being done, the lip was turned up, and the section of the fibres of the myrtiform muscle given by means of an incision made by gliding along the maxillary bone a short distance. After this operation, the edges of the incision made upon the sides of the curvature, were brought together by means of a suture, and thus the lip was drawn down, the exuberant portion of the mucous membrane destroyed, and this organ enabled to regain its proper form. The whole was kept in its proper position by means of an apparatus made for the purpose by Mr. F. Martin.

No marring accident took place. The tenth day, the threads were withdrawn, the re-union of the parts was complete ; but the apparatus was continued on for some time longer.

It is two months since the operation, the lip has regularly shapen itself, and exactly covers the teeth now replaced in their proper situations, and can perform all the functions which it is called upon for siezing and holding aliment, and for the articulation of sounds.

The utterance of speech which before was almost impossible, or at least very troublesome and disagreeable, has been entirely changed as to its nature, and is now performed without difficulty.

This notice is peculiarly fit here, in consideration of the singular method employed in remedying the deformity of the lip, and its successful issue. But that which above all merits attention, is the immense benefit and improvement to the jaw (derived from the skill of Mr. Brewster) judged as incurable by other practitioners of the continent of Europe.

We notice it with so much the more pleasure as there is in this operation a manifest improvement if we compare its simple methods, easily to be applied, and powerful in their action, with ligatures and the extraction of the teeth, so often recommended and performed, as if ever in despair of a remedy sufficiently proper for an evil so afflicting, and unfortunately so common on account of the little care which many parents bestow in watching the second dentition of their children.

Signed, S. P. D. M. P.

## Figures and Tables

**Figure f1:**
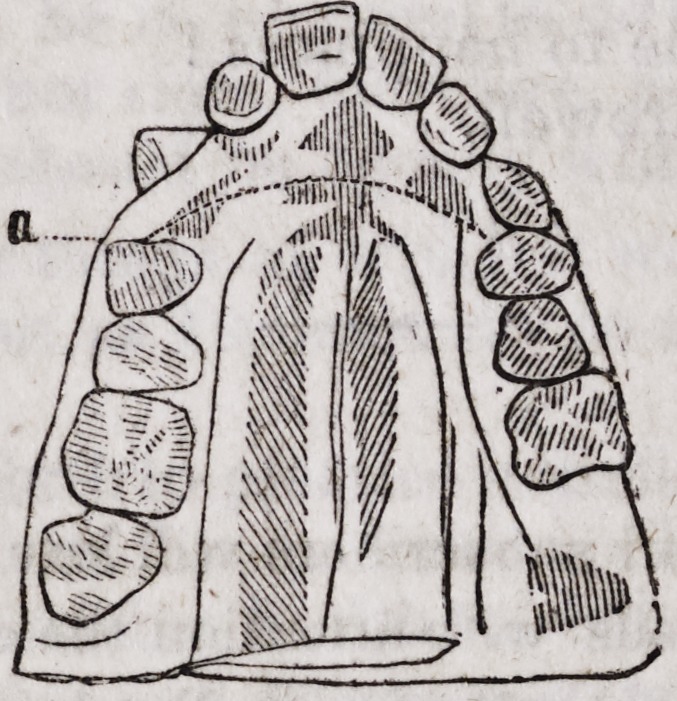


**Figure f2:**
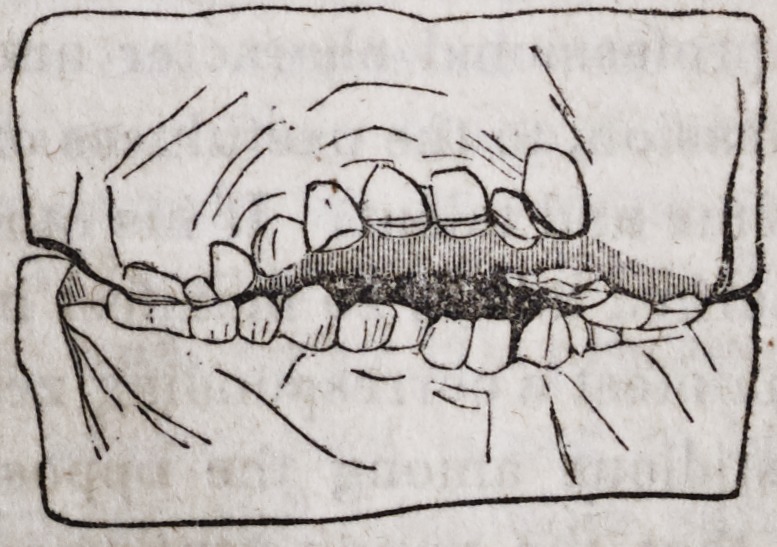


**Figure f3:**
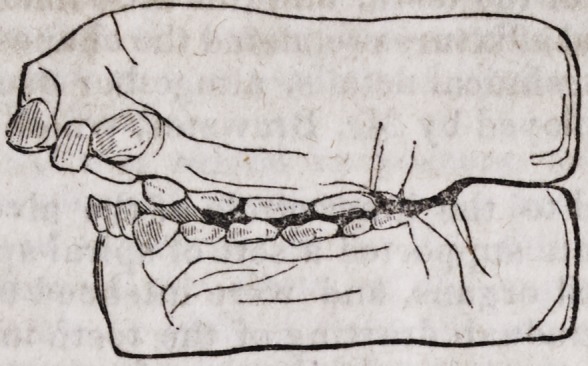


**Figure f4:**
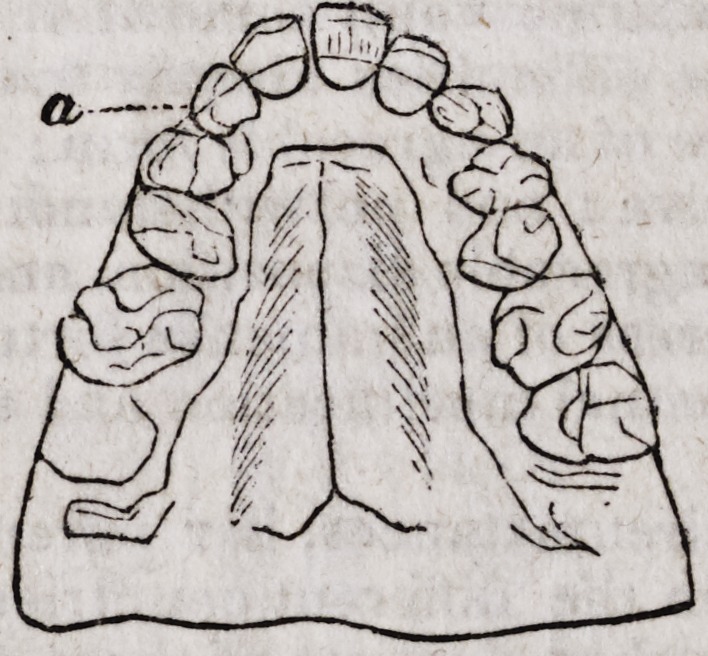


**Figure f5:**
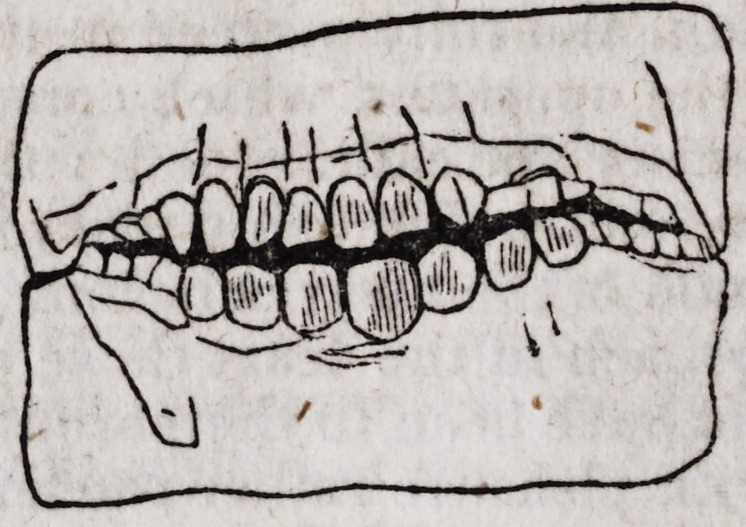


**Figure f6:**